# Differences in l-arginine metabolism and asthma morbidity among asthma patients with and without obstructive sleep apnea

**DOI:** 10.1186/s12931-022-02157-9

**Published:** 2022-09-05

**Authors:** Meghan D. Althoff, Guillermo Jimenez, Ryan Peterson, Ying Jin, Hartmut Grasemann, Sunita Sharma, Alex D. Federman, Juan P. Wisnivesky, Fernando Holguin

**Affiliations:** 1 Division of Pulmonary Sciences and Critical Care Medicine, University of Colorado Anschutz School of Medicine, 12700 East 19th Avenue, 9C03, Aurora, CO 80045 USA; 2 Department of Medicine, University of Colorado Anschutz School of Medicine, Aurora, CO USA; 3 Department of Biostatistics and Informatics, Colorado School of Public Health, University of Colorado Anschutz School of Medicine, Aurora, CO USA; 4 Division of Respiratory Medicine, Department of Pediatrics, Hospital for Sick Children, Toronto, Canada; 5 Division of General Internal Medicine, Icahn School of Medicine at Mount Sinai, New York, NY USA; 6 Division of Pulmonary, Critical Care, and Sleep Medicine, Icahn School of Medicine at Mount Sinai, New York, NY USA

**Keywords:** Asthma, Sleep apnea, Clinical epidemiology, Asthma epidemiology

## Abstract

**Background:**

Imbalance in l-arginine and nitric oxide (NO) metabolism has been implicated in the pathophysiology of asthma and obstructive sleep apnea (OSA), and both diseases impact the other’s morbidity. We sought to determine whether l-arginine/NO metabolism differs between adults with asthma with or without comorbid OSA, and its association with asthma morbidity.

**Methods:**

This is a cross-sectional study of 322 adults with asthma recruited in Denver, CO and New York City, NY. Data were collected on OSA status, spirometry, and metrics of asthma control and morbidity. l-Arginine metabolites were quantified in patient serum. Bivariate analyses and multiple regression were performed to determine differences between l-arginine metabolism, OSA and association with asthma morbidity.

**Results:**

Among the 322 participants, 92 (28.5%) had OSA. The cohort was 81.6% female, 23.4% identified as Black and 30.6% as Latino. Patients with asthma and OSA had significantly higher serum concentrations of NO synthase inhibitor asymmetric dimethylarginine (ADMA) (p-value = 0.019), lower L-arginine to ornithine ratios (p-value = 0.003), and increased ornithine (p-value = 0.001) and proline levels (p-value < 0.001) compared to those without OSA. In adjusted models, OSA was associated with worse asthma control, adjusted mean difference in asthma control questionnaire of 0.36 (95% confidence interval [CI]: 0.06 to 0.65), and asthma quality of life questionnaire, adjusted mean difference: − 0.53 (95% CI: − 0.85 to − 0.21), after adjusting for relevant covariates including body mass index and L-arginine metabolites.

**Conclusions:**

Adults with asthma and OSA had increased ADMA, an inhibitor of nitric oxide synthase, and greater metabolism of l-arginine via the arginase pathway compared to those with asthma alone, indicating a possible shared pathophysiological mechanism of these diseases.

## Background

Asthma and obstructive sleep apnea (OSA) are highly prevalent conditions, though the relationship between these diseases goes beyond their prevalence and shared risk factors [1]. Patients with asthma are more likely to have OSA than the general population [1, 2] and the relationship between OSA and asthma is thought to be bidirectional. Asthma patients are more likely to develop incident OSA, possibly due to airway and systemic inflammation associated with asthma [3]. Conversely, the intermittent nocturnal hypoxia and sleep fragmentation of OSA are thought to play a role in increasing asthma morbidity and severity. Concomitant OSA is associated with worse asthma control and increased asthma morbidity, including greater rates of emergency department visits and exacerbation rates [2–5].

The mechanisms behind the relationship between asthma and OSA are not understood. Asthma is a complex and heterogenous disease with multiple drivers of inflammation and activity. Dysregulation in the metabolism of l-arginine into downstream nitric oxide (NO) and l-citrulline by NO synthase (NOS) or urea and l-ornithine by arginase is thought to play an important role in asthma pathophysiology [6–8]. This dysregulation, however, appears to differ among asthma phenotypes. T2-driven asthma is characterized by elevated airway NO, which is produced by NOS isoforms including inducible NOS (iNOS) [3]. In obesity associated asthma, there is an imbalance of L-arginine and NOS inhibitor asymmetric dimethylarginine (ADMA) causing greater airway oxidative stress by uncoupling of iNOS leading to the formation of anion superoxide over NO [4, 9, 10]. Pilot studies have shown that supplementation with l-citrulline, via l-arginine recycling, can restore NO formation and improve lung function and asthma control [5]. l-Arginine can also be metabolized by arginases to form urea and l-ornithine, which is further metabolized to proline or polyamines, precursors of collagen deposition and cell proliferation, respectively. Arginase expression and activity is increased in asthma [6–8, 11] and obese asthma patients have been found to have increased expression of arginase compared to their lean counterparts [12].

Like asthma, OSA is associated with dysregulation of the L-arginine metabolism. OSA patients have been shown to have decreased L-arginine bioavailability, increased arginase activity, increased proline and decreased NO in small cross-sectional studies [13–16]. These studies, however, did not include individuals with comorbid asthma. Further supporting a component of shared pathophysiology, studies using continuous positive airway pressure (CPAP) to treat OSA among asthma patients found improvement in quality of life, asthma control, and spirometry [17–19]. Similarly, a small study of ten patients with asthma and OSA reported that treatment with nasal CPAP decreased ADMA and increased NO, implying that treatment of OSA may impact shared mechanisms of L-arginine metabolic dysregulation with asthma [20].

While CPAP therapy may improve asthma outcomes, CPAP adherence among OSA patients appears to be low [21]. A better understanding of the drivers of inflammation among asthma patients with OSA is necessary for the development of targeted therapeutic agents that would best improve asthma outcomes and morbidity in this population.

The objective of this study was to compare L-arginine metabolism for asthma patients with and without comorbid OSA, and to examine the impact of OSA on asthma morbidity after accounting for differences in l-arginine metabolism.

## Methods

### Study design and participants

We performed a cross-sectional analysis of baseline data from a cohort of asthma patients from New York City, NY and Denver, CO, collected between January 2017 and March 2020. Patients were recruited from primary care and pulmonary clinics associated with two large, urban hospitals with racially, ethnically and socioeconomically diverse catchment areas. Inclusion criteria were (1) patients ages 21–64 years (2) having an asthma diagnosis and actively using asthma medications, and (3) English or Spanish speaking. Participants were excluded if they had (1) concomitant diagnosis of chronic obstructive pulmonary disease or other chronic respiratory disease, (2) greater than or equal to 15 pack-year history of tobacco use and (3) diagnosis of dementia. Participants underwent an in-person interview, anthropomorphic measurements, baseline spirometry, and phlebotomy to obtain blood samples for the measurements of L-arginine metabolites. This study received human subjects research approval from the Colorado Multiple Institution Review Board (#16-1666).

### Clinical variables and outcomes

Presence of physician diagnosis of OSA was self-reported by participants. Participant height and weight were measured and used to calculate body mass index (BMI). BMI was categorized as underweight (< 18.5 kg/m^2^), normal (18.5–24.9 kg/m^2^), overweight (25.0–29.9 kg/m^2^) and obese (≥ 30 kg/m^2^) based on WHO criteria [22]. Other covariates of interest included age of onset of asthma, which was dichotomized to early onset, developing before 12 years of age, and late onset asthma, developing at or after 12 years of age. T2-high inflammation was defined as having either absolute eosinophil count greater than or equal to 200 and/or immunoglobulin E (IgE) levels greater than or equal to 200. These values were obtained from clinical records and were not directly measured as part of this study.

l-Arginine metabolites were quantified by Liquid chromatography with tandem mass spectrometry (LC–MS/MS): l-arginine, ADMA, l-citrulline, l-ornithine, and proline. The ratio of l-arginine to ornithine was calculated as it is a measure of l-arginine availability for NOS, and the l-arginine to ADMA ratio as it approximates NOS impairment [10, 23]. The absolute values were log-transformed for analysis. The total l-arginine availability index was defined as l-arginine/(l-citrulline + ornithine). A higher arginine availability index has been associated with fewer asthma exacerbations [24].

Clinical outcomes of interest were forced expiratory volume in one second (FEV_1_) and forced vital capacity (FVC), both measured by spirometry, asthma control as measured by Asthma Control Questionnaire (ACQ), asthma quality of life as measured by the mini-Asthma Quality of Life Questionnaire (AQLQ) and resource utilization measured by self-reported systemic corticosteroid use and emergency department (ED) visits in the past year. The ACQ and AQLQ are validated measures of asthma control and quality of life [25, 26]. Spirometry, most notably FEV_1_, is an objective, physiologic measurement of asthma activity. Spirometry was performed according to the American Thoracic Society (ATS) and European Respiratory Society (ERS) guidelines [27]. These outcomes were selected as they reflect a variety of aspects of asthma morbidity.

### Statistical analysis

Bivariate comparisons of demographics, l-arginine metabolites and outcomes among those with and without OSA were performed using nonparametric tests (Wilcoxon rank sum tests for continuous variables, Fisher’s Exact tests for binary/categorical variables). Multivariable linear regression models were used to assess the covariate-adjusted relationship between OSA and continuous outcome variables (ACQ, AQLQ, FEV_1_, FVC). A similar multivariable logistic regression model was used for the binary outcome of resource utilization (ED visit and/or steroid use in the past year). The BMI-adjusted relationships between OSA and logged l-arginine metabolites (and metabolite ratios) were assessed using multivariable linear regression, where coefficients were exponentiated to represent factor changes in expected outcomes holding BMI constant. All other multivariable models were adjusted for known confounders selected a priori: BMI, sex, age and race. These models were also adjusted for statistically significant biomarkers in the bivariate analysis to assess for independent effect the l-arginine metabolites. A small number of patients were missing metabolite data (n = 16; < 5%); these patients were excluded from analyses using the metabolite data. Analyses were performed using R version 4.0.2 [28].

## Results

### Study participants

Three hundred and twenty-two participants with asthma were enrolled in this study. Relevant demographics by OSA are described in Table 1. The majority of participants were female (81.6%). There was racial and ethnic diversity with 75 (23.4%) of participants identifying Black and 98 (30.6%) as Hispanic or Latino. Ninety-two participants (28.6%) reported a diagnosis of OSA. Those with OSA were more likely to be older with a median difference of 7 years (p < 0.001) and to have a higher BMI (median difference 7.2 kg/m^2^, p < 0.001). There was no difference in proportion of participants with T2-high inflammation among those with and without OSA (p = 0.137).

**Table 1:** Baseline demographic characteristics

	No OSA (N = 230)	OSA (N = 92)	Total (N = 322)
*Mean (SD)*			
Age^a^	44.3 (13.4)	53.2 (11.5)	46.9 (13.4)
BMI^a^	30.1 (8.6)	37.0 (9.2)	32.1 (9.3)
N (%)			
*Weight class*^a^			
Underweight	7 (3.1%)	0 (0.0%)	7 (2.2%)
Normal	55 (24.1%)	3 (3.3%)	58 (18.1%)
Overweight	71 (31.1%)	14 (15.2%)	85 (26.6%)
Obese	95 (41.7%)	75 (81.5%)	170 (53.1%)
Female sex^a^	192 (84.2%)	69 (75.0%)	261 (81.6%)
*Race*^a^			
Black/African American	51(22.4%)	24 (26.1%)	75 (23.4%)
Hispanic/Latino	65 (28.5%)	33 (35.9%)	98 (30.6%)
White	82 (36.0%)	27 (29.3%)	109 (34.1%)
Other	30 (13.2%)	8 (8.7%)	38 (11.9%)
*Recruitment site*^b^			
New York	130 (56.8%)	59 (64.1%)	189 (58.9%)
Colorado	99 (43.2%)	33 (35.9%)	132 (41.1%)
*Tobacco use*^c^			
Current	12 (5.3%)	3 (3.3%)	15 (4.7%)
Former	51 (22.4%)	27 (29.7%)	78 (24.5%)
Never	165 (72.4%)	61 (67.0%)	226 (70.8%)
*Asthma onset*^d^			
Early	88 (39.5%)	29 (31.9%)	117 (37.3%)
Late	135 (60.5%)	62 (68.1%)	197 (62.7%)
*Maintenance inhalers*			
ICS	80 (34.8%)	23 (25.0%)	103 (32.0%)
ICS/LABA	102 (44.3%)	57 (62.0%)	159 (49.4%)
LAMA	16 (7.0%)	10 (10.9%)	26 (8.1%)
*T2 inflammation*			
High	132 (57.4%)	44 (47.8%)	176 (54.7%)
Low	98 (42.6%)	48 (52.2%)	146 (45.3%)

ICS, inhaled corticosteroid; LABA, long-acting beta agonist; LAMA, long-acting muscarinic antagonist

^a^N = 320

^b^N = 321

^c^N = 319

^d^N = 314

### l-Arginine metabolites bivariate analysis

**Fig. 1:**
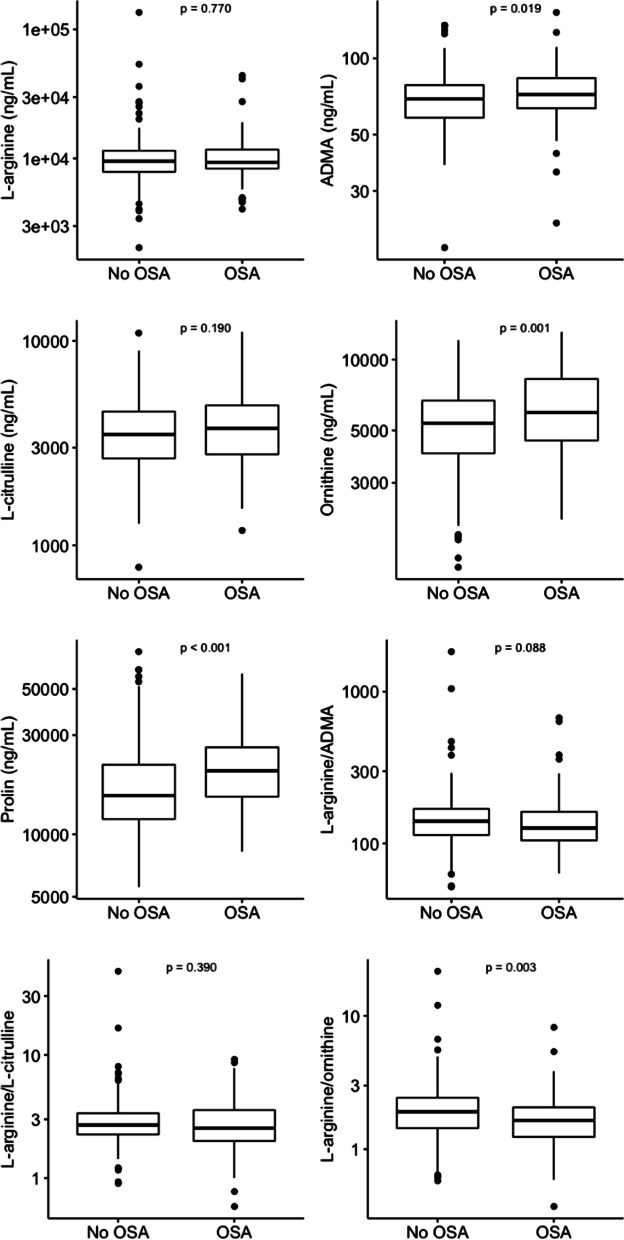
Bivariate associations between L-arginine metabolites and OSA

Levels of ADMA, ornithine, proline and the ratio of l-arginine to ornithine were statistically significantly different among asthma subjects with and without OSA (Fig. 1). Those with OSA had higher ADMA levels than those without OSA (median: 72 ng/ml versus 69.2 ng/nl, p = 0.019). The logged ratio of L-arginine/ornithine was lower among those with OSA (median: 0.50 versus 0.65, p = 0.003), and serum ornithine (median: 5960 ng/ml versus 5370 ng/ml, p < 0.001) and proline (20,200 ng/ml versus 15,360 ng/ml, p < 0.001) levels were increased, indicating increased metabolism of L-arginine via the arginase/ornithine pathway. In BMI-adjusted linear regression models for logged biomarkers, ornithine and proline levels remained statistically significantly higher among those with and without OSA (a 1.15 times higher expected ornithine level, 95% confidence interval [CI].: 1.04 to 1.27 and a 1.19 times higher proline level, 95% CI: 1.05 to 1.34).

### Association of OSA with Asthma Morbidity

Patients with OSA had greater odds of ED visits or oral steroid use than those without OSA (odds ratio [OR].: 2.00, 95% CI: 1.17 to 3.46) (Table 2). In bivariate comparisons, FVC, FEV_1_, ACQ and AQLQ were statistically different between the two OSA groups (p < 0.05 for all comparisons).

**Table 2:** Markers of asthma morbidity among participants with and without OSA

	No OSA N = 230	OSA N = 92	Total N = 322	*P-value*
Resource utilization	114 (50.0%)	60 (66.7%)	174 (54.7%)	0.009
*Mean (SD)*				
ACQ^a^	1.5 (1.0)	1.8 (1.2)	1.6 (1.1)	0.029
AQLQ^b^	5.4 (1.2)	4.8 (1.3)	5.2 (1.2)	< 0.001
FEV1 (L)^c^	2.5 (0.8)	2.2 (0.8)	2.4 (0.8)	0.026
FVC (L)^c^	3.3 (1.0)	3.0 (1.0)	3.2 (1.0)	0.047

ACQ: asthma control questionnaire, AQLQ: asthma quality of life questionnaire, FEV1: forced expiratory volume in one second, FVC: forced vital capacity

Higher ACQ scores indicate worse asthma control

Higher AQLQ scores indicate higher rated quality of life

^a^N = 316

^b^N = 312

^c^N = 300

In the adjusted analysis presented in Table 3, OSA was associated with worse asthma control as demonstrated by higher ACQ scores (adjusted mean difference [aMD].: 0.358, 95% CI: 0.064 to 0.652), and worse AQLQ scores (aMD: − 0.529, 95% CI: − 0.852 to − 0.207), with adjustment for age, sex, race, BMI, L-arginine/ADMA, proline, and the arginine availability index. After adjustment, the association of OSA with ED visits or steroid use was no longer significant (adjusted OR: 1.51, 95% CI: 0.83 to 2.81).

**Table 3:** Adjusted models of asthma morbidity

		Coefficient (95% CI)		OR (95% CI)	
	FEV1	FVC	ACQ	AQLQ	Resource utilization
Obstructive sleep apnea	− 0.01 (− 0.17, 0.16)	0.01 (− 0.19, 0.2)	0.36 (0.06, 0.65)	− 0.53 (− 0.85, − 0.21)	1.51 (0.83, 2.81)
Age	− 0.03 (− 0.03, − 0.02)	− 0.03 (− 0.04, − 0.03)	0.00 (− 0.01, 0.01)	− 0.01 (− 0.02, 0.00)	1.01 (0.99, 1.03)
Female sex	− 0.86 (− 1.04, − 0.68)	− 1.20 (− 1.42, − 0.99)	0.24 (− 0.08, 0.56)	− 0.59 (− 0.94, − 0.24)	1.96 (1.02, 3.8)
Race^a^					
Black/AA	− 0.44 (− 0.63, − 0.25)	− 0.74 (− 0.96, − 0.52)	0.26 (− 0.08, 0.6)	− 0.52 (− 0.89, − 0.15)	1.65 (0.83, 3.28)
Hispanic/Latino	− 0.20 (− 0.37, − 0.03)	− 0.52 (− 0.72, − 0.32)	0.25 (− 0.06, 0.57)	− 0.65 (− 0.99, − 0.31)	1.74 (0.93, 3.27)
Other	− 0.15 (− 0.39, 0.09)	− 0.38 (− 0.66, − 0.10)	0.34 (− 0.08, 0.76)	− 0.48 (− 0.95, − 0.01)	0.93 (0.39, 2.15)
BMI	0.00 (− 0.01, 0.00)	− 0.01 (− 0.02, 0.00)	0.01 (− 0.01, 0.02)	0.00 (− 0.02, 0.01)	1.05 (1.02, 1.09)
L − arginine metabolites^b^					
Arginine:ADMA	− 0.01 (− 0.26, 0.24)	0.01 (− 0.28, 0.3)	− 0.23 (− 0.68, 0.22)	0.47 (− 0.01, 0.96)	0.59 (0.23, 1.46)
Proline	− 0.01 (− 0.17, 0.15)	0.01 (− 0.17, 0.19)	0.13 (− 0.15, 0.40)	− 0.21 (− 0.51, 0.09)	1.14 (0.65, 1.98)
Arginine availability index	− 0.23 (− 0.48, 0.01)	− 0.23 (− 0.52, 0.06)	0.22 (− 0.22, 0.66)	− 0.34 (− 0.82, 0.15)	1.81 (0.73, 4.56)

^a^Reference White

^b^log transformed

## Discussion

In a diverse cohort of asthma patients, we found that participants with OSA had increased asthma morbidity, independent of L-arginine metabolite profiles. Though not associated with asthma morbidity, asthmatics with OSA had higher serum levels of NOS inhibitor ADMA, reduced L-arginine availability for NOS (lower L-arginine to ornithine ratio), and increased arginase activity (elevated L-ornithine and proline) compared to those without OSA, though notably only L-ornithine and proline levels remained statistically significant after adjustment for BMI. While not associated with asthma morbidity, it is possible that the differences in L-arginine metabolites between OSA and non-OSA asthmatics plays a role in shared pathophysiology, which was not investigated by this study.

Our findings that OSA is associated with decreased quality of life and asthma control is consistent with prior work [29], even after adjusting for BMI and age, both of which have been well-documented to impact asthma control and quality of life [30, 31]. We found a significant relationship between OSA and asthma morbidity as measured by oral corticosteroid use or ED visit, but this association became statistically insignificant after adjusting for confounders. Other observational studies have shown increased asthma exacerbations among those with OSA,[32–35] though this relationship is not consistently seen across the body of literature [36].

This study adds to the understanding of OSA and asthma as it is the first study to compare L-arginine metabolism among asthma patients with and without OSA. Our observation of increased ADMA in asthma patients with OSA is consistent with prior studies of obesity associated asthma [9, 10] and among OSA patients [13–15], though has not been previously evaluated in patients with both comorbidities. Of note, however, this association did not remain statistically significant after adjustment for BMI. The association of increased serum L-ornithine and proline among asthma subjects with OSA remained significant after accounting for BMI. While an increase in serum proline was recently described in patients with mild-to-moderate OSA [16], this association has not previously been reported among asthma patients with OSA.

In addition to increased serum ADMA, we found that those with OSA and asthma had lower L-arginine to ornithine ratios and increased L-ornithine and proline. The decreased L-arginine to ornithine ratio suggests preferred metabolism of l-arginine by arginase vs NOS. L-ornithine, the product of arginase activity, is further metabolized to proline, a precursor of collagen deposition. This may ultimately lead to airway remodeling and subsequent decrease in lung function and poor asthma control. While increased ADMA and arginase had been described in asthma before, this study provides the first data suggesting an even greater increase in arginase activity in asthma patients with OSA. Of note, we measured serum levels of circulating L-arginine metabolites, which may be more informative in obesity and comorbid OSA but may not be an adequate representation of airway L-arginine metabolism.

In interpreting the results from this study, it is important to consider the following limitations. We conducted a cross sectional study and could not determine which condition, asthma or OSA, developed first in each patient. The main predictor of interest, OSA, was self-reported, which could have resulted in differential misclassification of under-reported or undiagnosed OSA, for example, women are more likely than men to underreport OSA[37] and those with mild OSA may be asymptomatic and unaware of their diagnosis [38]. Data on CPAP usage was not available and variability in the treatment of OSA may have affected L-arginine metabolites and asthma outcomes among this group. Study data were not collected to assess the relationship between OSA, L-arginine metabolites and asthma morbidity outcomes, therefore the study may have been underpowered for the analyses presented here. Similarly, participants were included if they self-reported a physician diagnosis of asthma and use of asthma maintenance inhalers, however objective testing including bronchodilator reversible obstruction on spirometry or methacholine challenge was not performed. This may have resulted in the inclusion of participants who do not, in fact, have asthma, particularly among those with comorbid obesity, who may have many causes of dyspnea. Other studies have found misdiagnosis to be as high as 33% [39], though notably less than half of these patients were diagnosed by a pulmonologist and the majority of our participants were recruited from pulmonary and asthma clinics.

Finally, five percent of) participants did not provide blood samples, therefore there were some missing data for L-arginine metabolites that could have introduced bias and further decreased statistical power. While this cohort had significant racial and ethnic diversity, participants were predominantly female, limiting some of the external generalizability and impacting our ability to detect sex-specific differences.

Despite these limitations, this is the first study to show differences in L-arginine metabolism among asthma patients with and without OSA. The finding of increased L-arginine metabolism by arginase demonstrated by the reduction in L-arginine to ornithine ratio and increase ornithine and proline levels may provide potential therapeutic options for management of asthma in patients with comorbid OSA. The finding of increased ADMA is concordant with studies on obesity. What is not clear based on our results, is whether the increases seen in serum L-ornithine and proline is secondary to increased arginase expression and activity or whether this is secondary to increased channeling of L-arginine through arginase secondary to NOS inhibition by increased ADMA.

The mechanisms driving the relationship between OSA and asthma are not well defined, though there is likely a reciprocal relationship whereby each disease worsens control of the other. Independently, patients with OSA or asthma have both been found to have increased levels of ADMA, lower L-arginine bioavailability, and increased arginase activity [9, 10, 13–15]. In OSA patients, the imbalance between ADMA and L-arginine, leading to NOS uncoupling, is thought to contributed to pulmonary vasculature dysfunction via vascular oxidative stress [40] whereas in asthma this imbalance is thought to lead to increased airway oxidative stress. Whether this imbalance arises first in one disease then worsens or leads to the development of the other is unknown and needs to be answered in longitudinal studies. The increase in proline among those with OSA seen in our study suggests that OSA may cause an increase in proline, which may lead to long-term vascular and/or airway remodeling.

Therapeutic interventions that may result in increasing L-arginine availability for NOS include arginase inhibition and l-arginine or l-citrulline supplementation. A recent study on L-citrulline supplementation has shown improvement in lung function and asthma control among those with obesity associated asthma [5]. Future studies that assess OSA severity, treatment and compare non-asthma OSA patients to these groups would help better understand differences in L-arginine and nitric oxide metabolism in these disease states. A better understanding of the mechanisms of asthma morbidity among patients with concomitant OSA will help drive therapeutic discovery.

## Conclusions

We find that asthma patients with OSA have worse asthma control and quality of life compared to those without. Patients with asthma and OSA appeared to have increased systemic ADMA levels and arginase activity compared to those without OSA, indicating a possible shared mechanism between these diseases.

## Data Availability

One of the primary aims for the R01 that funded this cohort is still undergoing analysis, therefore the data from this project are not publicly available at this time.

## Abbreviations

OSA: Obstructive sleep apnea
NO: Nitric oxide
NOS: NO synthetase
iNOS: Inducible NO synthetase
ADMA: Asymmetric dimethyl arginine
CPAP: Continuous positive airway pressure
BMI: Body mass index
FEV1: Forced expiratory volume in one second
FVC: Forced vital capacity
ACQ: Asthma Control Questionnaire
AQLQ: Mini-Asthma Quality of Life Questionnaire
ED: Emergency department
OR: Odds ratio
CI: Confidence interval
